# Patient-Reported Quality of Life 6 Years After Breast Cancer

**DOI:** 10.1001/jamanetworkopen.2024.0688

**Published:** 2024-02-29

**Authors:** Maria Alice Franzoi, Antonio Di Meglio, Stefan Michiels, Emma Gillanders, Catherine Gaudin, Anne Laure Martin, Ines Vaz-Luis

**Affiliations:** 1 Cancer Survivorship Group, INSERM Unit 981, Molecular Predictors and New Targets in Oncology, Gustave Roussy, Villejuif, France; 2 Department for the Organization of Patient Pathways, Gustave Roussy, Villejuif, France; 3 Service de Biostatistique et d’Epidémiologie, Oncostat Inserm U1018, Université Paris–Saclay, Equipe Labellisée Ligue Contre le Cancer, Gustave Roussy, Villejuif, France; 4 UNICANCER, Paris, France

## Abstract

This cohort study assesses quality-of-life trajectories up to 6 years after breast cancer diagnosis among individuals in France.

## Introduction

Cancer contributes greatly to the global burden of chronic illness and has a tremendous impact patient’s quality of life (QOL), including physical, emotional, and social domains.^1^ The impact that cancer and its treatment have on an individual’s health trajectory can vary substantially, meaning that some patients require more care resources than others.^2^

Previous work^3^ focused on a group of patients with early stage I to III breast cancer (BC) treated with adjuvant chemotherapy identified a cluster of patients with poor initial QOL and subsequent severe, persistent postchemotherapy QOL deterioration up to 4 years after diagnosis. The current study aims to expand our previous work to unselected patients with BC to identify latent clusters of patients at risk for QOL deterioration up to 6 years after diagnosis and to assess the association of actionable host factors and health behaviors with QOL membership trajectory.

## Methods

This cohort study followed the STROBE reporting guidelines. We performed a longitudinal analysis of QOL using a large, national, prospective cohort in France (Chronic Toxicities Related to Treatment in Patients With Localized Cancer [CANTO])^4^ of patients with stage I to III BC treated from 2012 to 2018. This study was approved by French regulatory authorities and the French Committee for the Protection of Patients, and written informed consent was obtained from patients before participation.^4^

QOL (EORTC Quality of Life Questionnaire C30 summary score^3^) was assessed at diagnosis (baseline) and 1, 2, 4, and 6 years after diagnoses. Baseline clinical, sociodemographic, behavioral, tumor-related, and treatment-related characteristics were available. Trajectories of QOL and group membership associations were identified by iterative estimations of group-based trajectory models and multivariable multinomial logistic regression, respectively. Data were analyzed May 5, 2023, using SAS statistical software version 9.4 (SAS Institute), including the PROC TRAJ package, and R statistical software version 4.0.3 (R Project for Statistical Computing) with the MICE package. A 2-sided *P* < .05 was considered statistically significant.

## Results

Among 10 792 patients (mean [SD] age, 56.3 [11.2] years; 7982 [78.0%] with a partner; 5725 [57.3%] with monthly household income of <€3000; as of February 1, 2024, €1 = $1.09 US), 5695 received adjuvant chemotherapy and 8805 received adjuvant endocrine therapy. In the overall cohort, 4 QOL trajectory groups were identified: excellent (4934 participants [45.8%]), very good (3596 participants [33.3%]), deteriorating (1745 participants [16.1%]), and poor (517 participants [4.8%]) (Figure, panel A). Patients in the deteriorating trajectory group reported good baseline QOL (score, 73.3; 95% CI, 72.4-74.2), which significantly worsened at year 1 (score, 63.0; 95% CI, 62.1-63.9) and never recovered to pretreatment values through year 6 (score, 64.7; 95% CI, 63.3-66.0).

**Figure.**
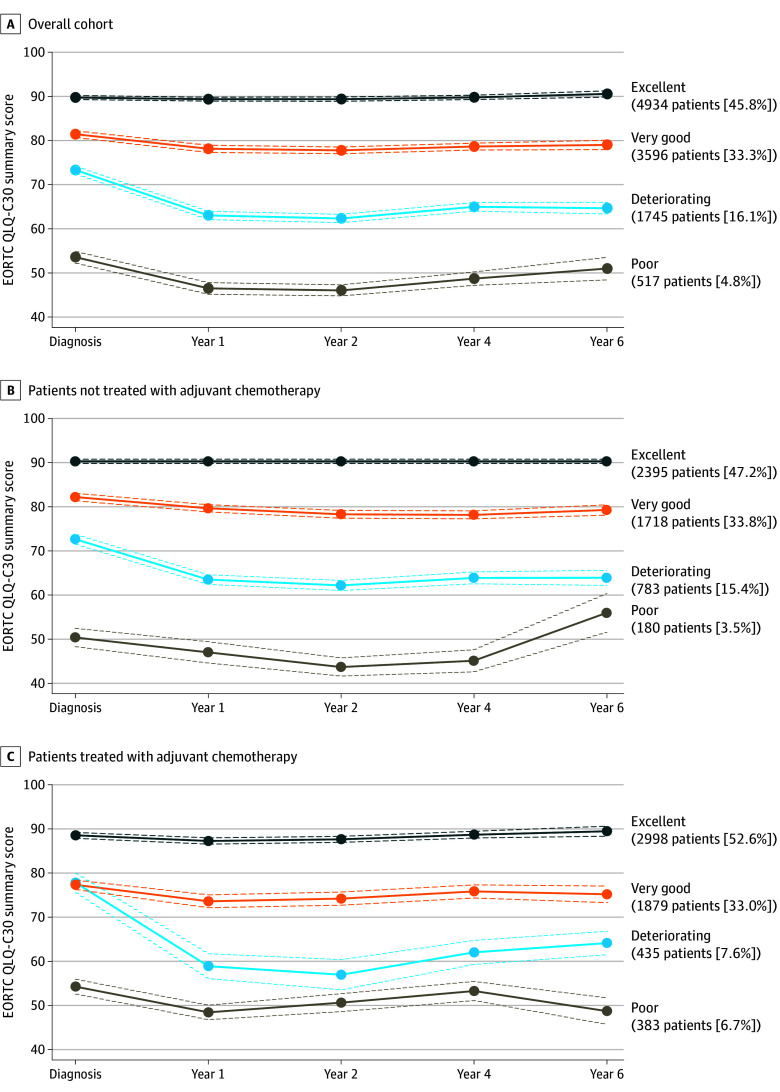
Trajectories of Quality of Life After Breast Cancer Graphs show EORTC Quality of Life Questionnaire (QLQ-C30) scores for all 10 792 patients (A), 5076 patients not treated with adjuvant chemotherapy (B), and 5695 patients treated with adjuvant chemotherapy (C). Dashed lines denote 95% CIs.

Common factors associated with membership in the deteriorating group in the overall cohort included younger age (adjusted odds ratio [aOR] for 10-year decrement, 1.10; 95% CI, 1.05-1.16), overweight (aOR vs lean, 1.90; 95% CI, 1.60-2.25), obesity (aOR vs lean, 1.25; 95% CI, 1.07-1.46), physical inactivity (aOR vs active, 1.13; 95% CI, 1.04-1.35), smoking behavior (aOR current vs never, 1.16; 95% CI, 0.98-1.36; former vs never, 2.14; 95% CI, 1.80-2.54), Charlson Comorbidity Index score 1 or greater (aOR vs 0, 1.56; 95% CI, 1.33-1.83), lower monthly household income (aOR for <€3000 vs ≥€3000, 1.50; 95% CI, 1.30-1.72), receipt of adjuvant chemotherapy (aOR vs no, 1.35; 95% CI, 1.14-1.59), and receipt of adjuvant endocrine therapy (aOR vs no, 1.46; 95% CI, 1.22-1.75) (Table). Independent analyses of factors associated with trajectory membership revealed associations similar to those found in the overall cohort among patients treated with and without adjuvant chemotherapy (Figure, panels B and C).

**Table.** Variables Associated With Quality of Life Trajectories in the Overall Cohort

Variable	Very good (n = 2865 [33.9%])		Deteriorating (n = 1312 [15.5%])		Poor (n = 383 [4.5%])	
	aOR (95% CI)^a^	*P* value	aOR (95% CI)^a^	*P* value	aOR (95% CI)^a^	*P* value
Age, continuous (10-y decrease)	1.10 (1.05-1.15)	<.001	1.14 (1.07-1.22)	<.001	1.20 (1.08-1.34)	<.001
Body mass index						
Overweight vs lean	1.42 (1.24-1.64)	<.001	1.90 (1.60-2.26)	<.001	2.19 (1.66-2.88)	<.001
Obesity vs lean	1.18 (1.05-1.33)	.005	1.25 (1.07-1.46)	.005	1.29 (0.99-1.68)	.06
Physical activity, sufficiently vs insufficiently active	0.86 (0.78-0.95)	.003	0.84 (0.74-0.96)	.01	0.75 (0.60-0.93)	.008
Smoking behavior						
Current vs never smoker	1.09 (0.97-1.23)	.14	1.16 (0.99-1.36)	.07	1.22 (0.93-1.60)	.15
Former vs never smoker	1.40 (1.22-1.62)	<.001	2.15 (1.81-2.55)	<.001	2.22 (1.69-2.93)	<.001
Alcohol behavior, daily vs less than daily	1.06 (0.91-1.22)	.45	0.99 (0.82-1.20)	.90	1.26 (0.93-1.70)	.14
Charlson Comorbidity Index score, ≥1 vs 0	1.24 (1.09-1.41)	.001	1.57 (1.34-1.84)	<.001	2.11 (1.65-2.69)	<.001
Marital status, partnered vs not	0.98 (0.86-1.11)	.72	1.01 (0.86-1.19)	.88	0.69 (0.54-0.88)	.003
Monthly household income, <€3000 vs ≥€3000^b^	1.14 (1.02-1.27)	.02	1.50 (1.30-1.73)	<.001	2.09 (1.62-2.70)	<.001
Breast cancer stage						
II vs I	1.07 (0.95-1.22)	.27	1.09 (0.95-1.29)	.30	1.18 (0.89-1.56)	.25
III vs I	1.07 (0.86-1.34)	.53	1.05 (0.79-1.40)	.72	1.26 (0.80-1.99)	.31
Breast cancer surgery, mastectomy vs partial breast surgery	1.07 (0.93-1.23)	.33	0.93 (0.78-1.11)	.41	0.96 (0.72-1.28)	.79
Axillary surgery, axillary dissection vs sentinel node	1.03 (0.90-1.18)	.66	1.07 (0.90-1.26)	.47	1.08 (0.82-1.44)	.58
Adjuvant chemotherapy, yes vs no	1.20 (1.06-1.36)	.005	1.35 (1.14-1.60)	<.001	1.43 (1.08-1.90)	.01
Adjuvant radiotherapy, yes vs no	1.15 (0.94-1.40)	.18	0.84 (0.65-1.08)	.18	0.99 (0.64-1.53)	.97
Adjuvant endocrine therapy, yes vs no	1.20 (1.06-1.38)	.005	1.47 (1.23-1.76)	<.001	0.99 (0.76-1.31)	.98
Adjuvant anti–human epidermal growth factor receptor–2 therapy, yes vs no	0.92 (0.78-1.08)	.30	1.08 (0.88-1.32)	.47	0.90 (0.64-1.27)	.55

Abbreviation: aOR, adjusted odds ratio.

^a^ aORs reflect results of multinomial regression for the overall cohort (8449 observations used; reference is excellent, 3889 observations [46.0%]).

^b^ As of February 1, 2024, €1 = $1.09 US.

## Discussion

This cohort study identified factors associated with QOL deterioration, including actionable psychosocial and lifestyle-related factors. Limitations of this study include some attrition in responding to the questionnaires over time and the fact that changes in behavior across time were not dynamically assessed. Nevertheless, our results could be used to facilitate the creation of personalized, proactive, and preventive supportive care pathways^1,4^ by targeting at-risk patients at diagnosis.^2^ The efficacy of such pathways to prevent QOL deterioration, as well as the best implementation and care delivery model, should be investigated.

## Notes

Supplement.
Data Sharing Statement

## References

[1] NCD Countdown 2030 Collaborators. NCD Countdown 2030: worldwide trends in non-communicable disease mortality and progress towards Sustainable Development Goal target 3.4. Lancet. 2018;392(10152):1072-1088. doi:10.1016/S0140-6736(18)31992-5 30264707

[2] Mayer DK, Alfano CM. Personalized risk-stratified cancer follow-up care: its potential for healthier survivors, happier clinicians, and lower costs. J Natl Cancer Inst. 2019;111(5):442-448. doi:10.1093/jnci/djy232 30726949 PMC6804411

[3] Di Meglio A, Havas J, Gbenou AS, . Dynamics of long-term patient-reported quality of life and health behaviors after adjuvant breast cancer chemotherapy. J Clin Oncol. 2022;40(27):3190-3204. doi:10.1200/JCO.21.00277 35446677 PMC9509127

[4] Vaz-Luis I, Cottu P, Mesleard C, . UNICANCER: French prospective cohort study of treatment-related chronic toxicity in women with localised breast cancer (CANTO). ESMO Open. 2019;4(5):e000562. doi:10.1136/esmoopen-2019-000562 31555487 PMC6735667

