# Supplementary material for: Patient-Reported Quality of Life 6 Years After Breast Cancer
Source: JAMA Netw Open. 2024 Feb 29;7(2):e240688. doi: 10.1001/jamanetworkopen.2024.0688 (PMC10905303; doi:10.1001/jamanetworkopen.2024.0688)
Supplement: Supplement. — Data Sharing Statement [file jamanetwopen-e240688-s001.pdf]

Data Sharing Statement

Franzoi. Patient-Reported Quality of Life 6 Years After Breast Cancer. *JAMA Netw Open*. Published February 29, 2024. doi:10.1001/jamanetworkopen.2024.0688

Data
Data available: No

Additional Information
Explanation for why data not available: Unidentified participant data may be available upon request of a scientific research proposal to Unicancer's Research and Development Department and CANTO's executive committee.
